# Patterns in US Cigar Sales and Prices from 2021 to 2025: An Examination of Pack Size, Flavor, “Natural” Descriptors, and Brand

**DOI:** 10.21203/rs.3.rs-9406409/v1

**Published:** 2026-04-14

**Authors:** Ollie Ganz, Nishi J. Gonsalves, Andrew A. Strasser, Ce Shang, Charity A. Ntansah, Olivia A. Wackowski, Michelle Jeong, Stefanie K. Gratale, Kymberle L. Sterling, Cristine D. Delnevo, Jessica King Jensen

**Affiliations:** Rutgers Institute for Nicotine & Tobacco Studies; Rutgers Institute for Nicotine and Tobacco Studies; Department of Psychiatry, University of Pennsylvania Perelman School of Medicine; Department of Internal Medicine, Ohio State University Wexner Medical Center; Rutgers Institute for Nicotine and Tobacco Studies; Rutgers Institute for Nicotine and Tobacco Studies; Rutgers Institute for Nicotine and Tobacco Studies; Rutgers Institute for Nicotine and Tobacco Studies; Rutgers Institute for Nicotine and Tobacco Studies; Rutgers Institute for Nicotine and Tobacco Studies; Rutgers Institute for Nicotine and Tobacco Studies

**Keywords:** tobacco, cigars, tobacco marketplace, flavored tobacco products

## Abstract

**Introduction:**

Cigars remain among the most affordable tobacco products in the U.S., with package features designed to increase appeal, including pack size, flavor, price, and potentially misleading descriptors, like “natural.” Building upon prior work showing frequent changes in the cigar market, we examined trends in US cigar unit sales and prices by package feature from 2021 to 2025.

**Methods:**

Nielsen Convenience Track national cigar sales data from 2021 to 2025 were used for analyses.

**Results:**

Total cigar unit sales decreased from $2.22 billion in 2021 to $1.79 billion in 2025, while average price per stick increased from $2.86 to $3.91. In 2025, the pack sizes with the greatest market share were two cigars/pack (46.8% of market) and single sticks (30.3%). In 2025, over one-quarter of cigar sales featured a “natural” descriptor; unflavored cigars had the greatest market share (43.3%), followed by flavored cigars (34.0%), and cigars with concept (i.e., ambiguous) descriptors (22.7%). From 2021 to 2025, market share decreased for flavored and unflavored cigars but increased for concept cigars. For average price per cigar, in 2025, two-packs, three-packs, and 20-packs were the cheapest per cigar compared with other pack sizes ($0.76, $0.75, and $0.66, respectively), with price per cigar for two- and three-packs decreasing from 2021 to 2025. Flavored cigars ($1.03) were cheaper than unflavored ($6.84) and concept cigars ($2.23) per cigar. Cigars with a “natural” descriptor were cheaper ($1.42) than those without ($4.60).

**Conclusions:**

Findings reflect a diverse cigar market with respect to pack size, flavor, and marketing descriptors.

## Introduction

Despite population-level declines in cigarette consumption in the United States (U.S.), cigar sales increased by 32% from 2009 to 2020.[1] This is significant considering that cigars pose similar health risks as cigarettes.[2, 3] Cigars are a diverse product class with multiple cigar types, including mass-merchandised cigarillos, little filtered cigars, large non-premium cigars, and premium cigars.[4] The most popular cigars are – compared to cigarettes – less expensive, available in many flavors, and frequently marketed as “natural,” features that may lend to their appeal, particularly among young people.[5–9] In 2022, the average price paid by consumers per pack of cigarettes in the U.S. was $8.55, while the average price paid per pack of cigars was only $1.66.[10] This price differential is largely due to the availability of small pack sizes (i.e., number of cigars sold within the pack, also referred to as “unit count” or “package quantity”) for cigars, since pack size is inextricably linked to price paid per pack. Unlike cigarettes, which in the U.S. must legally be sold with at least 20 cigarettes per pack, cigars are not subject to such restrictions and are therefore available in a variety of pack sizes. Notably, cigars are available in small pack sizes and at low out-the-door prices, for as little as $0.99 for a 2-pack or $1.00 for a 5-pack,[11–13] which increases affordability and may lower barriers to trial. Indeed, smaller packs, for which the out-the-door price is less than larger packs, may attract new consumers, including users of other brands, while larger pack sizes, which are typically cheaper *per cigar* compared with smaller packs, may attract those looking to get more value from their purchase.[14, 15]

Flavors also increase the appeal of cigars. Unlike cigarettes, for which flavors except menthol are banned, cigars are sold in a variety of flavors. Flavors mask the harshness of tobacco smoke, enhancing product appeal and reducing perceived harm, particularly among young people,[16] which may contribute to initiation, frequent consumption, and eventual transition to non-flavored use.[6, 17, 18] Additionally, cigars are marketed with concept descriptors that may imply flavor or may be unflavored. In 2020, characterizing flavored cigars comprised 53% of cigar sales in US convenience stores, while cigars with concept descriptors comprised 21.4% of the cigar market, and 26% were unflavored.[1, 16, 19, 20]

Research has documented the use of “natural” descriptors on cigar product packaging,[21, 22] and the impact of such descriptors on product appeal.[7] “Natural” descriptors can mislead consumers into thinking the product may pose reduced risk.[23–25] Notably, while the Family Smoking and Prevention Tobacco Control Act banned other descriptors implying reduced-risk (e.g., “light”, “mild”), it did not ban “natural.”[25, 26] Further, while the US Food and Drug Administration (FDA) later made an agreement with a prominent cigarette manufacturer to cease use of “natural” descriptors in their marketing,[27] similar restrictions do not extend to cigar marketing or packaging.

Delnevo et al. characterized the cigar market from 2009 to 2020 using convenience store sales data and observed notable changes over this period.[1] Market share for two- and three-packs of cigars grew from 2.3% to 42.6%, making them the most common pack size in 2020,[1] while single cigars and five-packs decreased in market share from 34.7% to 22.4% and 39.9% to 26.6%, respectively.[1] Sales of flavored cigars, specifically for those with fruit and sweet/candy flavors, also increased.[1] Additionally, studies have noted an increase in the sale of cigars with “natural” descriptors, with both market share and the number of unique cigar products with “natural” descriptors increasing from 2017 to 2021.[21] These findings reflect a cigar marketplace that is in constant flux and demonstrate how cigar manufacturers capitalize on the lack of federal and state cigar regulations by routinely changing package features, like pack size and flavor, to increase product appeal and sales. Building on findings from Delnevo et al.[1] the goals of this paper were to identify recent changes in the cigar marketplace using national convenience store data to document US cigar sales and average price per cigar from 2021 to 2025. Specifically, we documented cigar sales and per-cigar pricing overall and by pack size, flavor descriptors, “natural” descriptors, and brand.

## Methods

### Data source

Nielsen Convenience Track national cigar sales data from 2021 to 2025 were purchased for analyses. The Nielsen system uses in-store barcode readers and retail audits to produce sales estimates representative of convenience stores. Nielsen assigns product attributes, including pack size, brand, and flavor, and annual sales at the Universal Product Code (UPC) level. Items identified as “Wraps” were removed from the dataset for our analyses, as these products are typically intended for cannabis consumption and may not include tobacco.

## Measures

### Market Characteristics

Unit sales. Unit sales are defined as how the product is sold to the consumer. For example, a single cigar and a 5-pack of cigars would each count as one unit.

Market share. Market share was calculated based on the proportion of unit sales for a given product compared to total unit sales. For example, market share for flavored cigars was calculated by dividing flavored cigar sales by total cigar sales and multiplying by 100.

Price per cigar. Nielsen provides total annual dollar sales and total annual unit sales for each UPC. We calculated price per cigar by 1) dividing total dollar sales by total unit sales to determine total price per unit, then 2) dividing total price per unit by pack size. A small proportion (< 1%) of UPCs had implausibly high prices per cigar during initial review (e.g., $2 for a filtered cigar); for these items, we conducted a secondary review of the product descriptors provided by Nielsen to update the pack size variable and recalculate price per cigar as needed.

## Package features

Pack size. Pack size was coded using data provided by Nielsen on the number of cigars contained within a unit sold (e.g. two count). For UPCs that were cartons, which contain multiple units of cigars, pack size was coded to reflect the total number of cigars within the carton. For example, a carton containing ten, 20-packs of cigars was categorized as a 200-pack, since that is how the product was purchased. The least popular pack sizes were collapsed resulting in the following pack size codes: single-stick, 2 cigars/pack, 3 cigars/pack, 5 cigars/pack, 20 cigars/pack, 200 cigars/pack, and other (e.g., 4 cigars/pack, 6 cigars/pack). Pack sizes in the other category had 2% market share or less each year.

Flavor. Based on the flavor descriptor variable provided in the Nielsen data, all cigars were coded as unflavored, flavored, or concept.[28] Cigars were coded as unflavored if the Nielsen descriptor indicated that the product was unflavored or had descriptors related to tobacco flavor such as “original” or “pure tobacco.”[28] Flavored cigars contained an explicit characterizing flavor in the description (e.g., grape, rum) and were assigned to the following categories: fruit, sweet, wine, mint/menthol/ice, and other (e.g., beverages, alcohol, spices).[1] When flavor descriptors contained more than one flavor category (e.g., banana rum), we used a hierarchical approach to assign one flavor type to each product to better capture marketing categories:[29] mint/menthol/ice; wine; other (e.g., spice, alcohol); sweet; and fruit. For example, “strawberry cheesecake” was coded as “sweet,” since cheesecake falls into the “sweet” category and is above fruit (i.e., strawberry) in the hierarchy; while the flavor descriptor does contain a fruit component, it is classified sweet due to it being marketed as a dessert with a fruit descriptor.

Cigars were coded as concept if they contained a descriptor that did not indicate a specific taste (e.g., “jazz” and “silver”).[30] Since prior research shows that some products with concept descriptors are indeed flavored,[28, 31] we individually reviewed all concept flavors and further coded as either “concept flavored” or “concept unflavored”. The review process consisted of examining product websites and online cigar retailers (e.g., JR Cigars) to assess imagery and descriptions that implied a characterizing flavor. When a clear determination could not be made, we coded the product as “concept unflavored” to provide conservative estimates of flavored products. Since some concept descriptors were used across brands (e.g., Garcia Y Vega “Green” and Dutch Masters “Green”), we reviewed descriptors by brand.

“Natural” descriptor. UPCs that contained “natural,” “NTR,” or “NTRL” in the Nielsen item or product claim descriptions were coded as having a “natural” descriptor, with the remainder coded as not having a “natural” descriptor.

Brand. We examined unit sales for the following top-selling brands using a Nielsen-provided variable: Black & Mild, Swisher Sweets, Backwoods, Garcia Y Vega (including Game cigars), Dutch Masters, and White Owl. All other brands were coded as “other.”

## Statistical analyses

We determined the total unit sales and market share for each year (2021 to 2025), and sales by pack size, flavor, presence of a “natural” descriptor, and brand. Although prior studies examining cigar sales with Nielsen data have used dollar sales,[1, 32, 33] we chose to examine unit sales, which is a more accurate measure of consumption. Since price per cigar and per pack are tied to pack size, and therefore dollar sales, unit sales allowed us to understand market share by pack size in a way that was not driven by price differences, providing insight into consumer purchasing behavior. We also estimated the price per cigar for each year and by pack size, flavor, “natural” presence, and brand. Price variables were adjusted for inflation (in 2025 dollars).[34]

Additional analyses examined flavor and brand by pack size and “natural” descriptors. For these additional analyses by pack size, we focused on the most prevalent pack sizes from 2025 (i.e., singles, two-packs, five-packs) and 20-packs, since 20-packs are almost exclusively filtered cigars, which are typically used as very cheap cigarette substitutes and therefore warrant further investigation. Analyses were conducted using SAS software V.9.4.[35] This study was not considered human participant research and was exempt from IRB review.

## Results

In 2021, there were 3,178 unique cigar UPCs. Of these, there were 24 unique pack sizes, 301 unique flavors, 456 UPCs with natural descriptors, and 211 unique brands. In 2025, there were 3,595 unique cigar UPCs. Of these, there were 25 unique pack sizes, 354 unique flavors, 742 UPCs with natural descriptors, and 232 unique brands.

## Cigar unit sales and market share: 2021 to 2025

Overall, cigar units sold decreased by 19.6% from 2021 (2,224,230,925 units) to 2025 (1,787,787,569 units; Table 1).

## Pack size

In 2025, two-packs of cigars had the highest market share (46.8%), followed by single cigars (30.3%). These patterns were consistent throughout 2021 to 2025 (Table 1). Single cigars and 20-packs had higher market shares of unflavored cigars, while two-packs and five-packs had higher shares of flavored cigars (see Supplemental Tables).

### Flavor

In 2025, unflavored cigars had the highest market share (43.3%), followed by flavored cigars (34.0%) and concept cigars (22.7%). Among flavored cigars, sweet (39.6%) and fruit (37.7%) captured the greatest market share. From 2021 to 2025, the overall market share for unflavored (46.8% to 43.3%) and flavored (35.2% to 34.0%) cigars slightly decreased, while market share for concept cigars increased (18.1% to 22.7%). Sweet-flavored cigars showed the greatest growth in market share over the study period, from 34.1% to 39.6%.

Among cigars with concept descriptors in 2025, most were unflavored (Table 3). However, the market share for concept unflavored cigars decreased from 90.2% in 2021 to 82.6% in 2025, while concept flavored cigars increased from 9.8% in 2021 to 17.1% in 2025.

“Natural” descriptor

Market share for cigars containing a “natural” descriptor increased from 24.6% in 2021 to 27.6% in 2025. The number of unique UPCs sold with a “natural” descriptor increased 62.7% (from 456 to 742), compared to a 4.8% increase in products without “natural” descriptors. In 2025, most units with “natural” descriptors (67.8%) were two-packs, with flavors (46.6%) or concept descriptors (34.8%), and from Garcia Y Vega (47.0%), Dutch Masters (21.8%), and Backwoods (15.1%).

### Brand

The brands with the highest market share were Black & Mild (33.9%), Swisher Sweets (21.4%), and Garcia Y Vega (13.5%). From 2021 to 2025, Black & Mild had the largest increase in market share (30.4% to 33.9%); Swisher Sweets had the largest decline (25.8% to 21.4%).

3.2 Average Price Per Cigar from 2021 to 2025

Overall, the average price per cigar increased from 2021 ($2.86) to 2025 ($3.91; Table 2).

### Pack size

In 2025, single cigars were most expensive per cigar at $9.66 per cigar on average, and 20-pack cigars were the least expensive at $0.66 per cigar (Table 2). Price per cigar for two- and three-packs decreased from 2021 ($0.87 and $0.80, respectively) to 2025 ($0.76 and $0.75). Across all pack sizes in 2025, flavored cigars and concept cigars were less expensive than unflavored cigars (Supplemental Tables).

### Flavor

The average price per cigar increased for unflavored ($5.06 to $6.84 per cigar) and concept ($1.04 to $2.23 per cigar) cigars, while the average price per cigar for flavored cigars remained steady from 2021 to 2025 ($1.04 to $1.03). In 2025, among flavored cigars, “other” flavors were the most expensive ($2.17 per cigar) and fruit was the least expensive ($0.77 per cigar).

“Natural” descriptor

In 2025, the average price per cigar for cigars sold with “natural” descriptors was $1.42 compared to $4.60 per cigar for those without “natural” descriptors (Table 2 and Supplemental Tables).

### Brand

In 2025, Swisher Sweets had the cheapest average price per cigar, at $0.72 per cigar, while “other” cigar brands were most expensive, at $5.94 per cigar. All cigar brands (except “other”) were cheaper on average per stick in 2025 compared to 2021.

## Discussion

This study presents market share and prices for cigars sold in the U.S. from 2021 to 2025 using Nielsen convenience store sales data, across the following packaging features: pack size, flavor, “natural” descriptors, and brand. Overall, cigar *unit* sales declined, while the average price per cigar increased. While we cannot determine temporality with the present data, others have identified that increases in price are associated with decreases in cigar sales.[36] This decrease in sales may also have been influenced by the COVID-19 pandemic, as fluctuations in cigar sales were observed from 2020–2021.[37] Further, store closures during this time may have led to increased prices for cigars to compensate for decreasing unit sales.

Market share across pack sizes remained relatively stable over time, with singles and two-packs dominating the market across the study period. This is consistent with 2012–2020 sales data reported by Delnevo et al., although they did not distinguish between two- and three-packs.[1] Our study extends this work by separately examining these pack sizes. Further, an analysis of packaging features for top-selling cigar products in 2018 found that over 90% of the top-selling cigars were sold as singles or two-packs.[22]

Generally, there was a negative association between pack size and average price per cigar, with 20-packs sold as cheaply as $0.66 per cigar. This association is widely documented and highlights the trade-off between a low out-the-door price of small packs versus the price per cigar savings of larger packs.[14, 38] This is consistent with studies on other tobacco products, which have found that prices per cigarette are lower for cartons versus individual packs, and that e-liquids with greater volume have lower prices per mL. [39] Smaller cigar packs, which are cheaper overall than larger pack sizes, are particularly appealing to young people, who are price sensitive.[40] In fact, two- and three-packs became more affordable over the study period, with the average price per stick decreasing from 2021 to 2025 for both pack sizes, after adjusting for inflation. Single cigars were by far the most expensive on average per cigar, which is likely driven by premium cigars. Despite representing a small proportion of the market, they are significantly more expensive and more likely to be purchased as singles compared with other cigar types.[4] The “other” brand category is also likely largely comprised of premium cigar brands, contributing to the category’s high average price per stick; this was the only brand category that increased, rather than decreased, in average price per stick across the study period. An inherent challenge of cigar research is that there are no set legal definitions of the distinct and diverse array of cigar types, which have distinct consumer profiles and smoking patterns.[4] Therefore, there is no straightforward way to distinguish between cigar types in the Nielsen data or other data sources, creating challenges for surveillance of cigar sales and patterns of use.

Unflavored cigars had the highest market share, followed by flavored cigars and concept cigars. Among flavored cigars, market share increased for wine- and sweet-flavored cigars, while market share for other flavors either remained steady or declined. These findings generally align with prior published sales data.[1, 33] However, while Delnevo et al. observed an increase in market share for sweet-flavored cigars from 2009–2020, they also observed a decrease in sales of wine cigars during this time period.[1] Although unflavored cigars had the greatest market share, it is worth noting that about one-third of cigars sold during the study period were explicitly flavored. Further, there was an increase in the number of unique cigar flavors sold from 2019 to 2025 (227 to 354).[41] With the withdrawal of the proposed FDA product standard to ban characterizing flavors in cigars in 2025,[42] the availability of flavored cigars and the trend toward an increased number of flavors available will likely persist. This raises public health concerns given that flavored cigar use is disproportionately prevalent among young people, particularly non-Hispanic Black youth,[43, 44] and compared to non-flavored cigar use, flavored cigar use is associated with daily smoking and greater nicotine dependence among adults.[45]

Although cigars with concept descriptors had lower market share than other cigars, overall unit sales and market share for cigars with concept descriptors increased over the study period (18.1% to 22.7%). This was driven by an increase in concept descriptors implying flavor or marketed to suggest flavor, which offset a decrease in concept descriptors without any flavor indicators (i.e., concept unflavored cigars). By design, it is unclear whether cigars with concept descriptors are explicitly flavored,[30] which introduces challenges for regulators, particularly in localities and states where flavored tobacco products are banned, because enforcement cannot rely on explicit flavor terms alone.

This study provided new data documenting the substantial prevalence of “natural” descriptors, used to market 27.6% of cigars sold in the last two years, contributing to the limited research in this area. While the market share of packs featuring “natural” descriptors was relatively stable across the period, there was a 56.6% increase in the number of unique cigar products sold with a “natural” descriptor, suggesting a trend towards increased use of “natural” marketing and greater availability of cigars with “natural” descriptors. We identified a lower price per cigar among products with versus without “natural” descriptors, potentially driven by a higher use of “natural” marketing among cheaper, mass-merchandise cigars. Indeed, the “natural” descriptor was largely featured on two-packs, and as others have noted,[22] were primarily used by two brands that are popular for their mass-merchandise cigarillo products, Garcia Y Vega and Dutch Masters. Our finding that most cigars with “natural” descriptors were flavored and on small pack sizes is troubling, as the combination of both flavor and “natural” descriptors on an inexpensive product may lead to a synergistic effect on reducing perceptions of harm and increasing product appeal, especially among young people, and warrants future research examining these effects.

This study has limitations. Convenience store data from Nielsen do not capture online sales or sales from other retailer types, including specialty tobacco shops and informal markets. Our findings underestimate US cigar sales and prevalence of some features, as some data indicate online sales are disproportionately for larger pack sizes.[46] However, convenience stores account for many tobacco retailers[47] and are where the majority of mass-merchandise cigarillos and filtered cigars are purchased by consumers.[4] Additionally, flavor categorizations have inherent limitations. Flavor profiles can vary greatly within a category (e.g., Chocolate and Nectar Sweet were both categorized as sweet).[30] Additionally, while we made efforts to categorize cigars with concept descriptors as flavored or unflavored, we were not able to do this for all cigars, and it is unclear if our categorizations align with consumer perceptions of whether the product is flavored or not.

Our findings reflect a diverse cigar market with respect to pack size, flavor, and marketing descriptors. The diversity of pack sizes and flavors is in sharp contrast to the cigarette marketplace, which is more stringently regulated than the cigar market. Our study shows that small pack sizes continue to dominate the cigar market. These small pack sizes are sold cheaply and are often flavored, raising concerns about their appeal to young people. Our study also observed a high proportion of cigars with “natural” descriptors, which may lead to misperceptions of harm. Policy responses such as minimum pack size requirements, restrictions on characterizing flavors, and regulations on the use of suggestive descriptors should be considered. Since cigar manufacturers are likely to continue to capitalize on the relatively limited federal regulation of cigars by modifying packaging features to increase appeal, study findings may add to the growing body of evidence that can inform future cigar product packaging regulations.

## Supplementary Material

Supplementary Files

This is a list of supplementary files associated with this preprint. Click to download.

- SupplementalTables2.13.26.docx

## Figures and Tables

**Table 1:** Cigar Unit Sales Market Share by Pack Size, Flavor, Natural Descriptor, and Brand, Nielsen U.S. Convenience Store Data, 2021–2025

	2021	2022	2023	2024	2025
	% Market Share	% Market Share	% Market Share	% Market Share	% Market Share
**Overall Units (N)**	2,224,230,925	2,209,336,291	2,036,350,684	1,895,792,888	1,787,787,569
**Pack Size**					
Single cigar	30.4	30.5	31.5	30.1	30.3
2 cigars per pack	49.9	50.1	46.2	48.2	46.8
3 cigars per pack	5.6	5.7	6.4	7.6	7.8
5 cigars per pack	9.9	9.9	9.4	9.9	10.8
20 cigars per pack	1.8	1.7	4.3	1.6	1.6
200 cigars per pack	0.1	0.1	0.1	0.1	0.1
Other	2.3	2.0	2.1	2.5	2.6
**Flavor Descriptor**					
Unflavored	46.8	47.1	46.9	44.8	43.3
Flavored	35.2	34.9	33.6	33.6	34.0
*Among Flavored*					
Fruit	42.8	44.1	40.4	38.5	37.7
Sweet	34.1	32.7	34.1	35.6	39.6
Wine	10.3	10.6	14.8	16.4	13.7
Mint/Menthol/Ice	1.3	1.2	1.3	1.5	1.5
Other	11.5	11.3	9.5	8.0	7.2
Concept	18.1	18.0	19.5	21.7	22.7
**Natural Descriptor**					
No	75.4	72.9	73.8	72.4	72.4
Yes	24.6	27.1	26.2	27.6	27.6
**Brand**					
Black & Mild	30.4	30.8	33.1	33.1	33.9
Swisher Sweets	25.8	25.5	23.6	22.7	21.4
Backwoods	5.2	4.8	4.1	4.6	4.8
Garcia Y Vega	12.0	14.6	14.1	13.6	13.5
Dutch Masters	6.9	7.5	7.0	6.6	6.1
White Owl	8.7	7.5	6.9	6.3	6.1
Other	10.8	9.5	11.2	13.1	14.2

Note: Unflavored descriptors include explicit tobacco flavor descriptors. “Other” flavor descriptors include alcohol, spices, and beverages. Garcia y Vega includes Game cigars.

**Table 2:** Average Price Per Cigar by Pack Size, Flavor, Natural Descriptor, and Brand, Nielsen U.S. Convenience Store Data, 2021–2025

	2021	2022	2023	2024	2025
	USD $	USD $	USD $	USD $	USD $
**Average Price Per Cigar ($)**	2.86	3.02	3.11	3.56	3.91
**Pack Size**					
Single cigar	7.01	7.38	7.49	8.87	9.66
2 cigars per pack	0.87	0.81	0.84	0.82	0.76
3 cigars per pack	0.80	0.77	0.73	0.76	0.75
5 cigars per pack	1.21	1.33	1.24	1.42	1.35
20 cigars per pack	0.34	0.34	0.36	0.42	0.66
200 cigars per pack	0.13	0.12	0.13	0.12	1.13
Other	1.17	1.37	1.55	1.59	1.78
**Flavor Descriptor**					
Unflavored	5.06	5.39	5.41	6.47	6.84
Flavored	1.04	0.99	1.06	1.01	1.03
*Among Flavored*					
Fruit	0.85	0.79	0.87	0.78	0.77
Sweet	0.97	0.92	0.93	0.92	0.89
Wine	1.39	1.12	1.04	0.99	0.92
Mint/Menthol/Ice	0.85	0.59	0.71	0.71	0.79
Other	1.97	2.09	2.13	2.00	2.17
Concept	1.04	1.02	0.89	0.94	2.23
**Natural Descriptor**					
No	3.15	3.41	3.58	4.16	4.60
Yes	1.28	1.25	1.21	1.27	1.42
**Brand**					
Black & Mild	1.31	1.20	1.14	1.39	1.08
Swisher Sweets	0.93	0.77	0.74	0.76	0.72
Backwoods	1.65	1.89	1.72	1.64	1.57
Garcia Y Vega	1.09	1.01	0.99	0.95	0.94
Dutch Masters	1.19	1.04	0.99	1.01	0.92
White Owl	0.94	0.89	0.85	0.82	0.80
Other	4.08	4.50	4.69	5.31	5.94

Note: USD: US Dollar ($). Unflavored descriptors include explicit tobacco flavor descriptors. “Other” flavor descriptors include alcohol, spices, and beverages. Garcia y Vega includes Game cigars. Average price per cigar for 2021–2024 were adjusted for 2025 inflation.

**Table 3:** Flavored and Unflavored Unit Market Share for Cigars with Concept Descriptors, Nielsen 2021–2025

Total Concept Units, N	2021	2022	2023	2024	2025
	401,734,904	398,010,425	396,659,312	411,083,612	400,039,639
Concept Unflavored	90.2	89.1	85.4	81.6	82.6
Concept Flavored	9.8	10.9	14.6	18.4	17.1

## Data Availability

Data from Nielsen cannot be shared.

## IMPLICATIONS

The FDA and state- and local-level policymakers have the authority to regulate cigar products. Findings regarding the availability of small pack sizes, cigars with a “natural” descriptor, and flavored and concept cigars call attention to policy responses such as minimum pack size requirements, restrictions on characterizing flavors, and regulations on the use of suggestive descriptors.
